# Host-specific differences in top-expanded TCR clonotypes correlate with divergent outcomes of anti-PD-L1 treatment in responders versus non-responders

**DOI:** 10.3389/fimmu.2023.1100520

**Published:** 2023-03-27

**Authors:** Jessy John, Samantha M. Y. Chen, Rachel A. Woolaver, Huaibin Ge, Monika Vashisht, Ziyu Huang, Zhangguo Chen, Jing H. Wang

**Affiliations:** ^1^ UPMC Hillman Cancer Center, Division of Hematology and Oncology, Department of Medicine, University of Pittsburgh, Pittsburgh, PA, United States; ^2^ Department of Immunology and Microbiology, University of Colorado, School of Medicine, Aurora, CO, United States; ^3^ UPMC Hillman Cancer Center Biostatistics Facility, University of Pittsburgh, Pittsburgh, PA, United States; ^4^ Department of Immunology, University of Pittsburgh, Pittsburgh, PA, United States

**Keywords:** immune checkpoint inhibitor (ICI), T cell receptor sequencing, TCR repertoire, individualized anti-tumor immune responses, head and neck squamous cell carcinoma (HNSCC)

## Abstract

Immune checkpoint inhibitors (ICIs) have revolutionized cancer treatment; however, the responses to ICI treatment are highly variable in different individuals and the underlying mechanisms remain poorly understood. Here, we employed a mouse squamous cell carcinoma (SCC) model where tumor-bearing recipients diverged into responders (R) versus non-responders (NR) upon anti-PD-L1 treatment. We performed in-depth TCRβ sequencing with immunoSEQ platform to delineate the differences in CD8 tumor-infiltrating lymphocytes (TILs). We found that R and NR CD8 TILs both exhibited evidence of clonal expansion, suggesting activation regardless of response status. We detected no differences in clonal expansion or clonal diversity indexes between R vs. NR. However, the top expanded (>1%) TCRβ clonotypes appeared to be mutually exclusive between R and NR CD8 TILs, showing a preferential expansion of distinct TCRβ clonotypes in response to the same SCC tumor in R vs. NR. Notably, the mutual exclusivity of TCR clonotypes in R vs. NR was only observed when top TCRβ clonotypes were counted, because such top-expanded clonotypes are present in the opposite outcome group at a much lower frequency. Many TCRβ sequences were detected in only one recipient at a high frequency, implicating highly individualized anti-tumor immune responses. We conclude that differences in the clonal frequency of top TCR clonotypes between R and NR CD8 TILs may be one of the factors underlying differential anti-PD-L1 responses. This notion may offer a novel explanation for variable ICI responses in different individuals, which may substantially impact the development of new strategies for personalized cancer immunotherapy.

## Introduction

Immune checkpoint inhibitors (ICIs) have emerged as a promising therapy used to treat different types of cancers including head neck squamous cell carcinoma (HNSCCs) (1–4). However, the response rate of HNSCCs to ICI treatment remains relatively low when applied alone as monotherapy or combined with chemoradiation (3). The underlying mechanisms for such highly variable responses remain incompletely understood (5, 6). Prior studies have focused on the differences in tumor-intrinsic factors such as tumor mutational burden (TMB) or PD-L1 expression, or environmental factors such as microbiome. For example, TMB has been reported to correlate to ICI response in melanoma and non-small cell lung cancer (NSCLC) (7–9). However, it remains controversial whether the level of TMB correlates to ICI response in HNSCC. Prior studies showed that TMB^high^ HNSCCs responded to ICI treatment better (10–12), whereas conflicting data showed that TMB did not correlate to ICI response (13, 14). One biomarker for correlating ICI responses in HNSCCs is PD-L1 expression. The clinical trial data showed that the PD-L1 expression is predictive of the response rate and survival if the combined positive score (CPS) was used, which considers PD-L1 expression from both tumor and TME (3). However, the PD-L1 expression based on the tumor proportion score (TPS) does not predict ICI response rate or survival (3). Nonetheless, ICI responses cannot be accurately predicted by CPS of PD-L1 expression; thus, additional biomarkers are needed. Taken together, these studies indicate that tumor-intrinsic differences may not fully explain differential ICI responses in HNSCCs.

We hypothesized that immunological heterogeneity in the host may also contribute to the differential responses to ICI treatment (5, 6). One of the most influential factors underlying immunological heterogeneity is the adaptive immune system including T and B cells. Most conventional T cells are αβ T cells and each individual T cell expresses a unique T cell antigen receptor (TCR) consisting of an alpha (α) and a beta (β) chain. Both TCRα and TCRβ chains are generated *via* a somatic DNA recombination process, termed V(D)J recombination. TCRs can be grouped into distinct “clonotypes” containing TCRα and/or TCRβ chains encoded by unique V(D)J gene segments and complementarity-determining region 3 (CDR3). CDR3 is of particular importance because it encompasses the highly divergent junction of V(D)J recombination and determines antigen specificity of TCRs.

Prior studies showed that the level of CD8 tumor-infiltrating lymphocytes (TILs) correlate positively with HNSCC outcomes (15–18). Given the critical role of CD8 T cells in anti-tumor immunity, we employed a mouse SCC model, where tumor-bearing recipients diverged into responders vs. non-responders upon anti-PD-L1 treatment, to examine the contribution of CD8 T cells to differential responses to ICI (19). In line with prior studies, we showed that CD8 T cells were required for the efficacy of anti-PD-L1 treatment. Moreover, we showed that top expanded TCR clonotypes were almost mutually exclusive in the CD8 TILs of responders vs. non-responders (19). We previously examined the TCR repertoire using single-cell TCR sequencing (19, 20), which can simultaneously detect both TCRα and TCRβ chains; however, the sequencing depth of this method is rather limited. Hence, in the current study, we employed the immunoSEQ platform to perform in-depth sequencing of TCRβ clonotypes in the CD8 TILs of responders and non-responders. Our data suggest that differences in the clonal frequency of top TCR clonotypes between responder and non-responder CD8 TILs may be one of the factors underlying differential anti-PD-L1 responses.

## Method

### *In vivo* mouse work and tumor injection

A223 tumor line was described previously (21). Tumor cells were injected into wild-type (WT) C57BL/6 (B6) (Stock no. 000664) (Jackson Laboratories). Both male and female mice (6-8 weeks) were used for the study. All mice were maintained under specific pathogen-free conditions in the vivarium facility of University of Colorado Anschutz Medical Campus (Aurora, CO) or in the UPMC Hillman Cancer Center Animal Facility (Pittsburgh, PA). Animal work was approved by the Institutional Animal Care and Use Committee of University of Colorado Anschutz Medical Campus (AMC) (Aurora, CO) and University of Pittsburgh (Pittsburgh, PA).

A223 cells were cultured and trypsinized as described earlier (19). A223 cells (1×10^5^) were resuspended to a final volume of 100μL in 50% Matrigel (Corning)/50% PBS and injected subcutaneously into one flank of each mouse. Tumor length and width were measured with calipers, and tumor volume was calculated as (length×width^2^)/2. When tumor size reached ~250-350mm^3^, tumor-bearing mice were treated with anti-PD-L1 (200µg/mouse/time diluted in PBS, clone 10F.9G2, BioXCell, Catalog# BE0101) *via* intraperitoneal (i.p.) injection three times (2-day interval) or PBS only as vehicle control (control group). Of note, as shown in our previous study (19), all the tumor-bearing mice in the control group had tumor growing out, and no substantial differences were observed in tumor growth among control group. To assess treatment effects, relative change in tumor volume (RCTV) was calculated as the change in tumor volume (TV) from the start of treatment (TV_0_) to the TV at day n (the endpoint of control group) (TV_n_) divided by TV_0_ (RCTV=[TV_n_−TV_0_]/TV_0_). For example, if tumor-bearing mice were treated on day 12 (the start of treatment), day 14, and day 16 with anti-PD-L1, and tumors were collected and analyzed on day 20, the RCTV would be calculated as follows: RCTV= (TV_day20_−TV_day12_)/TV_day12_. Based on the RCTV, anti-PD-L1 treated recipients were divided into responders (RCTV<0), slow progressors (0<RCTV ≤ 1.5) and non-responders (RCTV>1.5).

### DNA sequencing of CDR3 regions in the TCRβ chains of TIL samples

Tumors were harvested from 3 R (1R-3R) and 4 NR (4NR-7NR) tumor-bearing mice and single-cell suspensions were prepared as described previously (19). Single-cell suspensions were subjected to EasySep™ Mouse CD8a Positive Selection Kit II (StemCell Technologies, Catalog#18953) according to manufacturer’s instructions to purify CD8 T cells followed by genomic DNA isolation. DNA concentration and purity were determined by NanoDrop™ One^C^ (Thermo Fisher Scientific). Genomic DNA samples were submitted to Adaptive Biotechnologies for amplification and sequencing of TCRβ chain CDR3 regions using the ImmunoSEQ platform (Adaptive Biotechnologies) at survey resolution (22). The same amount of genomic DNA was used for all 7 samples. The TCRβ chain CDR3 sequences and corresponding V, D, and J segments were delineated using the algorithm developed by ImMunoGeneTics (IMGT) collaboration (23). Data were analyzed using ImmunoSEQ Analyzer platform (http://www.adaptivebiotech.com/immunoseq/analyzer) and also extracted for further analysis using R (version 4.1.0) or using immunarch (version 0.6.7) package of R. Only in-frame TCR rearrangements were used for clonotype analysis. Details of the samples are included in **Supplemental Table 1**.

Clonal expansion index was calculated by dividing the clonal frequency of each clonotype in top-20 expanded clonotypes by the sum (∑) of all singleton clonotype frequency, $\frac{\%\;in\;each\;sample}{\sum_{i=1}^{n}singleton\%\;in\;each\;sample\;}$ where n is the total number of singleton clonotypes. Singleton clonotype was defined as a given clonotype identified only once in a given sample. Simpson clonality is the square root of the sum over all observed rearrangements of the square fractional abundances of each rearrangement (Simpson clonality = $\sqrt{\sum_{i=1}^{n}P_{i}^{2}}$ where n is the total number of rearrangements, ‘*i’* is each rearrangement and *P_i_* is productive frequency of rearrangement ‘*i’*). Simpson clonality is also the square root of Simpson’s D and is robust across differences in sampling depths. Simpson’s D (also known as Simpson’s dominance index) is the sum over all observed rearrangements of the square fractional abundances of each rearrangement (Simpson’s D = $\sum_{i=1}^{n}P_{i}^{2}$ ). 

Normalized TCR Richness is calculated by dividing the number of unique productive TCR sequences with the number of total productive TCR sequences.

### Statistical analysis

Data were presented as mean ± SEM. Statistical significance was calculated with unpaired t test or Fisher’s Exact test (24). Analysis was performed using GraphPad Prism version 9.3.1 for Windows (GraphPad Software).

## Results

### Clonal expansion of CD8 TILs in responders vs. non-responders

**Figure 1:**
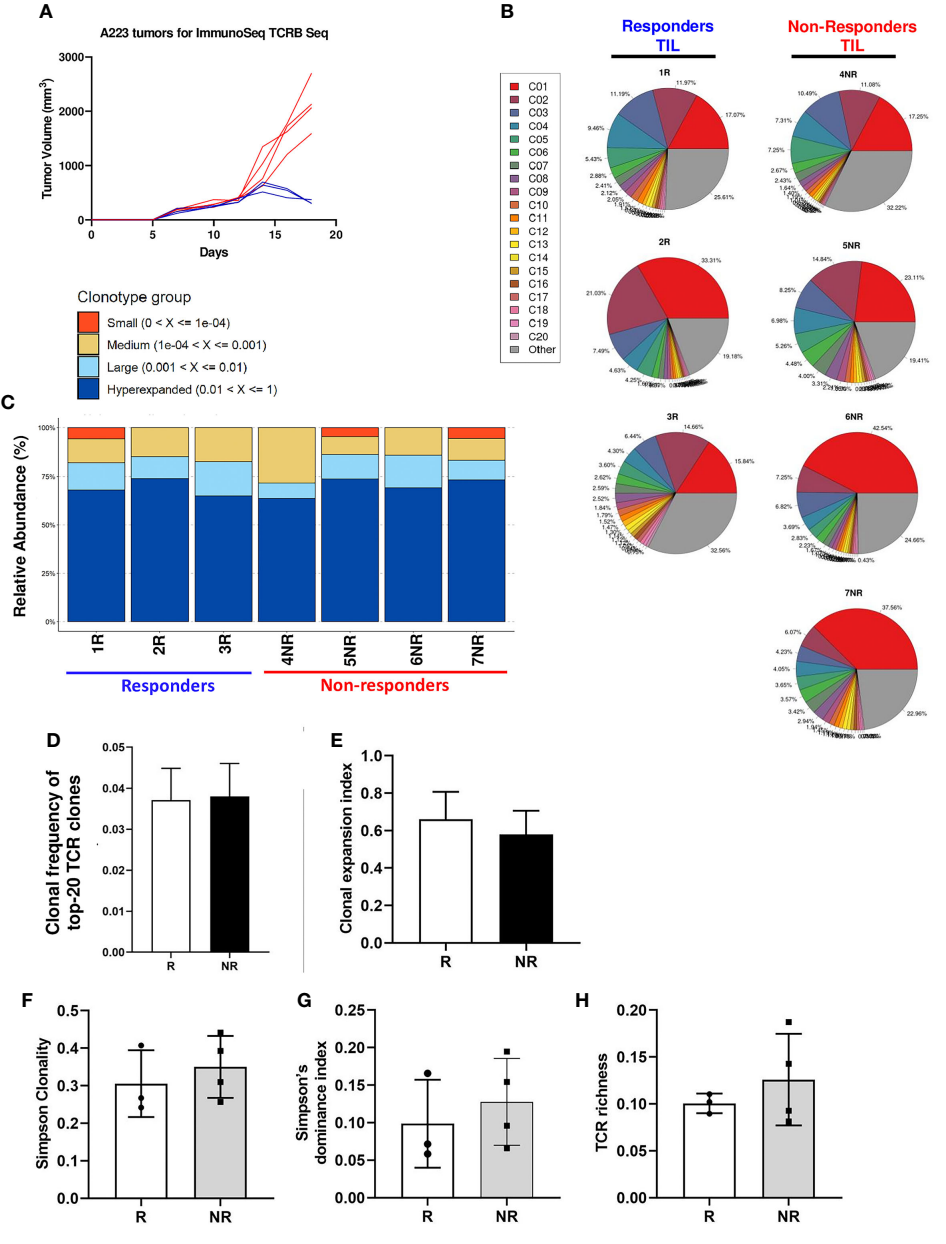
Clonal expansion in responder and non-responder CD8 TILs. **(A)** Tumor growth curves of R (blue) and NR (red) mice. CD8 T cells were isolated from the tumors of R (n=3, 1R, 2R, 3R) and NR (n=4, 4NR, 5NR, 6NR, 7NR) mice after anti-PD-L1 treatment on day 18 after tumor inoculation followed by gDNA extraction for ImmunoSEQ TCRβ sequencing. **(B)** TCRβ clonal expansion in 7 sequenced TIL samples. Each pie chart shows the percent of top 20 TCRβ clonotypes in each sample. A single-colored pie slice represents the percentage of cells containing the same TCRβ clonotype in the entire sample, and the gray slice represents the combined percentage of remaining TCRβ clonotypes in each sample. **(C)** Relative abundance of TCRβ clonotypes in 7 sequenced CD8 TIL samples. The relative abundance of individual TCRβ clonotypes was calculated using repClonality function in immunarch package and TCR clonotypes were grouped accordingly as small, medium, large, and hyperexpanded. **(D)** Average of clonal frequency of top-20 TCRs in R (n=3 mice, N=60 clonotypes in total) vs. NR (n=4 mice, N=80 clonotypes in total). **(E)** Clonal expansion index between R vs NR CD8 TILs. **(F-H)** No differences in TCR diversity indexes between R vs. NR CD8 TILs, including Simpson clonality **(F)**, Simpson’s dominance index **(G)**, and normalized TCR richness **(H)**.

One limitation of single-cell TCR sequencing is that only a few thousand cells were analyzed. To further test our findings of mutual exclusivity of TCR clonotypes in R vs. NR, we performed TCRβ CDR3 DNA sequencing using Adaptive Biotechnologies’ immunoSEQ platform, which allowed us to examine much more productive TCRβ CDR3 sequences. In total, we sequenced 3 responders (1R, 2R, 3R) and 4 non-responders (4NR, 5NR, 6NR, 7NR) by isolating CD8 TILs from 7 individual mice that were treated with anti-PD-L1 (**Figure 1A**). The total numbers of sequenced templates and productive rearrangements were shown for all 7 samples (**Supplemental Table 1**).

Consistent with single-cell TCR sequencing data, we found that CD8 TIL samples underwent clonal expansion to a similar extent in R vs. NR groups (**Figure 1B**). To better delineate the relative abundance of all TCR clones in the entire repertoire, we also employed repClonality function of immunarch package to analyze our TCRβ sequencing data. We found that the vast majority of TCRβ clonotypes in CD8 TIL samples belonged to hyperexpanded or large clones (**Figure 1C**), demonstrating clonal expansion regardless of R or NR state. We showed the top 10 TCR clonotypes (including VDJ usage and CDR3 sequences of TCRβ) for all 7 samples and their abundance in each sample (**Supplemental Table 2**).

To better delineate the clonal expansion status of R vs. NR CD8 TILs, we compared the average clonal frequency of the top 20 expanded clonotypes between R vs. NR samples (N=60 for R vs. N=80 for NR). Our data showed no differences in the average clonal frequency of top 20 clonotypes (**Figure 1D**). The number of productive TCR templates varied significantly in different samples due to sampling variation (**Supplemental Table 1**). In order to minimize the effects of sampling variation, we calculated “clonal expansion index” using a normalization method (See details in Method). Again, we did not identify any significant differences in clonal expansion index between R vs. NR CD8 TILs (**Figure 1E**). Taken together, we conclude that both R and NR CD8 TILs underwent clonal expansion to a similar extent.

### No differences in clonal diversity between responder vs. non-responder CD8 TILs

Diversity is an important global characteristic of TCR repertoires, which can be generally considered as the number of unique TCR (richness) and their relative abundances (evenness). Various metrics can be calculated to capture one or both properties of TCR diversity. These matrices can quantitatively characterize TCR repertories and the distribution patterns of TCR abundances in a T cell population, which include Simpson Clonality, Simpson’s D (also known as Simpson’s dominance index) and TCR richness. Simpson clonality is a method of quantifying the shape of a repertoire, ranging between 0 and 1, where values approaching 1 indicate a nearly monoclonal population. Simpson’s dominance index ranges from 0 to 1, where values approaching 0 correspond to a polyclonal, infinitely large, perfectly even repertoire and values approaching 1 correspond to a nearly monoclonal sample, where one clone dominates. TCR richness is defined as the number of unique productive TCR rearrangements in a given sample; however, this parameter can be influenced by sampling variation. Hence, we calculated the normalized TCR richness (See details in Methods). Overall, we found no significance differences in Simpson clonality (**Figure 1F**), Simpson’s dominance index (**Figure 1G**) or normalized TCR richness (**Figure 1H**) between R vs. NR CD8 TILs. We conclude that there is no significant difference in TCR diversity between R vs. NR CD8 TILs.

### Top TCR clonotypes appear to be mutually exclusive in R vs. NR CD8 TILs assessed by TCRβ DNA sequencing

**Figure 2:**
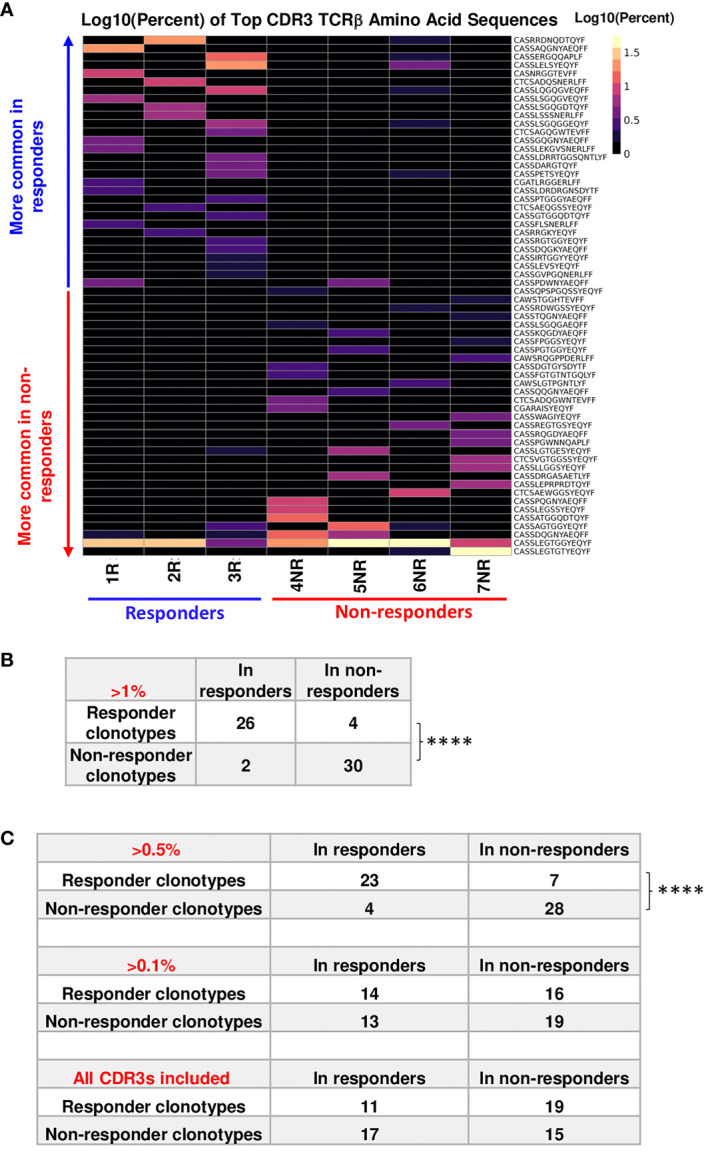
Top TCRβ clonotypes appear to be mutually exclusive between responder (R) and non-responder (NR) CD8 TILs. **(A)** Heatmap of top TCRβ clonotypes in all samples. Top TCRβ clonotypes (abundance >1% of a given sample) defined as having the same TCRβ CDR3 a.a. sequences were sorted by average abundance in R vs. average abundance in NR. **(B)** Statistical analysis for the mutual exclusivity of R vs. NR top TCRβ clonotypes. Top TCRβ clonotypes were assigned to R (n=30) or NR (n=32) group according to their overall abundance in either group. 26 of 30 R clonotypes were observed only in R and 28 of 32 NR clonotypes were observed in NR only, when the abundance of shared clonotypes is set to be >1% for the opposite outcome group. Statistical significance was calculated by Fisher’s Exact test (****, *P*<0.0001). **(C)** Differences between two groups were calculated by Fisher’s Exact test to evaluate the exclusivity of TCRβ clonotypes in R or NR. Top: 23 R clonotypes were observed only in R, whereas 7 R clonotypes were also observed in NR. 28 NR clonotypes were observed in NR only, whereas 4 NR clonotypes were also observed in R, when the abundance of shared clonotypes is set to be >0.5% in the entire repertoire of the opposite outcome group (****, *P*<0.0001). Middle: 14 R clonotypes were observed only in R, whereas 16 R clonotypes were also observed in NR. 19 NR clonotypes were observed in NR only, whereas 13 NR clonotypes were also observed in R, when the abundance of shared clonotypes is set to be >0.1% in the entire repertoire of the opposite outcome group (N.S., no significance). Bottom: 11 R clonotypes were observed only in R, whereas 19 R clonotypes were also observed in NR; 15 NR clonotypes were observed in NR only, whereas 17 NR clonotype was also observed in R, when all CDR3β sequences were counted for the opposite outcome group including clones less than< 0.1%.

In line with our findings from single-cell TCR sequencing (19), we showed that the commonly expanded TCRβ (defined as >1% of the entire repertoire of a given sample) CDR3 a.a. sequences appeared to be almost mutually exclusive between R (n=30) vs. NR (n=32) samples (**Figure 2A**). Designation to R vs. NR group was determined by average abundance in R vs. average abundance in NR (**Table 1**). To test if top TCRβ CDR3 a.a. sequences in R group are also observed in NR group and vice versa, we performed a Fisher’s Exact test (24) and found that top R TCRβ CDR3 sequences were much more frequently observed in R than in NR, and vice versa (*P*<0.0001). For instance, out of 30 R clonotypes, 26 of them were detected in R with a frequency of >1%, whereas only 4 of them were detected in NR with a frequency of >1% (**Figure 2B**; **Table 1**). When we expanded our analysis to clonotypes with a frequency of >0.5%, we still detected a significant difference between clonotypes in R vs. NR (**Figure 2C**, top, *P*<0.0001), namely, the clonotypes in R group occurred much more frequently in R than NR, and vice versa. However, when we counted the clones with a frequency of >0.1% in the opposite outcome group or all clones regardless of their clonal frequency in either group, we did not identify a statistical significance for a given top CDR3 sequence to be observed in R vs. NR group (**Figure 2C**, middle and bottom). Taken together, these data show that the top CDR3 sequences (>1%) in R group are also present in NR group, albeit at a much lower frequency (**Table 1**).

**Table 1:** The percentage (%) of each clonotype in each sample.

	TCRβ CDR3	1R	2R	3R	4NR	5NR	6NR	7NR
1	CASRRDNQDTQYF	0	21.15475	0.42523	0	0.366238	0.728988	0.072454
2	CASSAQGNYAEQFF	17.13624	0	0	0	0	0	0.006587
3	CASSERGQQAPLF	0	0	14.77169	0	0	1.114923	0.065867
4	CASSLELSYEQYF	0	0	15.94614	0	0	2.744425	0.250296
5	CASNRGGTEVFF	9.466213	0	0	0	0	0	0
6	CTCSADQSNERLFF	0	7.561945	0.151868	0	0.170343	0.042882	0.03952
7	CASSLQGQGVEQFF	0	0	6.4797	0	0	0.686106	0.03952
8	CASSLSGQGVEQYF	5.453755	0	0	0	0	0	0
9	CASSLSGQGDTQYF	0.007243	4.675082	0.121494	0	0.136275	0.085763	0.032934
10	CASSLSSSNERLFF	0	4.301075	0.060747	0	0.034069	0.214408	0.085628
11	CASSLSGQGGEQYF	0	0	4.323175	0	0	1.072041	0.065867
12	CTCSAGQGWTEVFF	0	0	3.604333	0	0	0.343053	0.026347
13	CASSGQGNYAEQFF	2.940537	0	0	0	0	0	0
14	CASSLEKGVSNERLFF	2.448034	0	0	0	0	0	0
15	CASSLDRRTGGSQNTLYF	0	0	2.632378	0	0	0.300172	0.01976
16	CASSDARGTQYF	0	0	2.551382	0	0	0.428816	0.006587
17	CASSPETSYEQYF	0	0	2.642503	0	0	0.557461	0.03952
18	CGATLRGGERLFF	2.136597	0	0	0	0	0	0
19	CASSLDRDRGNSDYTF	1.933802	0	0	0	0	0	0
20	CASSPTGGGYAEQFF	0	0	1.873038	0	0	0.042882	0.059281
21	CTCSAEQGSSYEQYF	0	1.694717	0.030374	0	0	0	0
22	CASSGTGGQDTQYF	0.036214	0.105189	1.822416	0.03951	0.008517	0.171527	0.28323
23	CASSFLSNERLFF	1.535453	0	0	0	0	0	0
24	CASRRGKYEQYF	0	1.496026	0.030374	0	0	0	0
25	CASSRGTGGYEQYF	0	0	1.498431	0	0	0	0
26	CASSDQGKYAEQFF	0	0	1.29594	0	0	0.042882	0.013173
27	CASSIRTGGYYEQYF	0	0	1.123823	0	0	0	0
28	CASSLEVSYEQYF	0	0	1.133948	0	0	0.343053	0
29	CASSGVPGQNERLFF	0	0	1.083325	0	0	0.300172	0
30	CASSPDWNYAEQFF	2.904324	0.280505	0.182242	0	3.347245	0.128645	0.013173
31	CASSQPSPGQSSYEQYF	0	0	0	1.007507	0	0	0
32	CAWSTGGHTEVFF	0	0	0	0	0	0	1.014359
33	CASSRDWGSSYEQYF	0	0	0	0	0	1.072041	0.032934
34	CASSTQGNYAEQFF	0	0	0	0	0	0	1.106574
35	CASSLSGQGAEQFF	0.166582	0.116877	0.030374	1.185302	0.136275	0.214408	0.013173
36	CASSKQGDYAEQFF	0	0	0.080996	0	1.371263	0	0
37	CASSFPGGSYEQYF	0	0	0	0	0	0.085763	1.192201
38	CASSPGTGGYEQYF	0.166582	0.140252	0.070872	0.098775	1.243506	0.471698	0.01976
39	CAWSRQGPPDERLFF	0	0	0	0	0	0.085763	1.455671
40	CASSDGTGYSDYTF	0.021728	0	0.040498	1.422363	0.170343	0.042882	0
41	CASSFGTGTNTGQLYF	0.050699	0	0	1.659423	0	0	0
42	CAWSLGTPGNTLYF	0	0	0	0	0	1.672384	0.065867
43	CASSQQGNYAEQFF	0	0	0.232864	0	2.231496	0	0.006587
44	CTCSADQGWNTEVFF	0.101398	0	0	2.42987	0	0	0
45	CGARAISYEQYF	0.217281	0	0	2.70644	0	0	0
46	CASSWAGIYEQYF	0	0	0	0	0	0	2.970623
47	CASSREGTGSYEQYF	0	0	0	0	0	2.87307	0.131735
48	CASSRQGDYAEQFF	0	0	0.010125	0	0	0.042882	3.458042
49	CASSPGWNNQAPLF	0	0	0	0	0	0	3.570017
50	CASSLGTGESYEQYF	0	0	0.546725	0	4.198961	0.385935	0
51	CTCSVGTGGSSYEQYF	0	0	0	0	0	0.042882	4.103544
52	CASSLLGGSYEQYF	0	0	0	0	0	0	4.274799
53	CASSDRGASAETLYF	0	0	0.31386	0	4.53113	0.25729	0.01976
54	CASSLEPRPRDTQYF	0	0	0	0	0	0.128645	6.105915
55	CTCSAEWGGSYEQYF	0	0	0	0	0	6.818182	0.098801
56	CASSPQGNYAEQFF	0.26798	0	0	7.388384	0	0	0
57	CASSLEGSSYEQYF	0.18831	0	0	7.368629	0	0	0.111975
58	CASSATGGQDTQYF	0.340407	0	0	10.62821	0	0.085763	0
59	CASSAGTGGYEQYF	0	0	1.275691	0	15.4331	0.77187	0.032934
60	CASSDQGNYAEQFF	0.528717	0	0.556849	11.22086	5.314709	0.25729	0.032934
61	CASSLEGTGGYEQYF	24.67589	33.4619	3.401843	17.40419	38.12282	54.6741	7.508892
62	CASSLEGTGTYEQYF	0	0	0	0	0	0.643225	37.6762

Blue, responder TCR clonotypes; Red, non-responder TCR clonotypes.

Out of 6907 productive rearrangements identified in 7 samples, only 5 TCRβ CDR3 a.a. sequences were shared among the 7 samples (**Supplemental Table 3**). The frequency of these 5 shared CDR3 sequences was very low except shared clonotype 1 (**Supplemental Table 3**). We compared commonly expanded TCRβ CDR3 DNA sequences between R vs. NR and found that they also appeared to be almost mutually exclusive (**Supplemental Figure 1**). Taken together, our data showed the mutual exclusivity of the top expanded TCRβ CDR3 sequences between R vs. NR, when high frequency clones were counted in the opposite outcome group. Of note, the top TCR clonotypes also differed in a recipient-specific manner within the same group (R or NR), suggesting a highly individualized anti-tumor immune response.

### Preferential usage of V or J gene segments in CD8 TILs of responders vs. non-responders

To obtain a broader overview of whether TCRβ features correlate with anti-PD-L1 response, we examined the usage of germline Vβ or Jβ gene segments in individual mice and between R vs. NR group. For each of the 7 samples sequenced for TCRβ, we showed the sum or average of productive rearrangement frequency for all TCR Vβ gene segments (**Supplemental Figures 2A, B**). To better compare the R vs. NR group, we separated the above two parameters into R (1R-3R) vs. NR (4NR-7NR) (**Supplemental Figures 2C, D**). By this comparison, we found a significant difference in the average of productive rearrangement frequency of TCRBV26-01 and TCRBV30-01 between R vs. NR (**Supplemental Figure 2E**).

Similar analysis was performed for TCR Jβ gene segments for all 7 samples (**Supplemental Figures 2F, G**) or R vs. NR samples (**Supplemental Figures 2H, I**). When we compared the average of productive rearrangement frequency for all TCR Jβ gene segments, TCRBJ01-04 and TCRBJ01-05 appeared to be more frequently used by R group; however, this difference was caused by drastic differences in a single R mouse (2R for TCRBJ01-04 and 3R for TCRBJ01-05) (**Supplemental Figure 2I**), consistent with a highly individualized anti-tumor immune response in different mice. Overall, we did not identify any preferential usage of certain Jβ gene segments that were significant between R vs. NR TILs.

## Discussion

Using a mouse SCC model, we found that tumor-bearing recipients diverged into R vs. NR upon anti-PD-L1 treatment. We performed TCRβ sequencing of CD8 TILs from R vs. NR group to identify differences in their TCR repertoires. We made a few unexpected discoveries (1): there were no differences in clonal expansion status or TCR diversity indexes between R vs. NR CD8 TILs (2); top expanded TCR clonotypes differed substantially, almost mutually exclusive, between R vs. NR CD8 TILs, when the threshold of clonal frequency was set as >1% or >0.5%. This observation is consistent with our previous single-cell sequencing results (19); (3) however, when clonotypes with lower frequencies were included, we failed to detect differences in TCR clonotypes between R vs. NR CD8 TILs, a finding not observed in our previous study (19). Basically, in the current study, we found that R clonotypes were also detected in NR recipients, albeit at a much lower frequency, and vice versa. Taken together, our current study suggests that differences in the clonal frequency of top TCR clonotypes between R and NR CD8 TILs may be one of the factors underlying differential anti-PD-L1 responses.

We previously examined the CD8 TILs from R vs. NR using singe-cell TCR sequencing, and our results showed that the top expanded TCR clonotypes appeared to be mutually exclusive between R and NR CD8 TILs (19). The major differences between the two sequencing platforms are as follows: single-cell TCR sequencing (10× genomics platform) uses a droplet technique which can capture the single T cells in a droplet and sequence the RNA of both TCRα and TCRβ chains from a given T cell, thus, providing detailed information of TCR clonotypes. However, the major limitation of single-cell TCR sequencing is that only a few thousand cells can be analyzed due to the technical design (maximal 10,000 cells loaded per run), and the prohibitive cost of each sample for single-cell sequencing approach. Hence, this technology has limited throughput to detect rare clones whose clonal frequencies are much lower. If the cost per cell can be further reduced for single-cell approach, it may allow substantially deeper profiling to increase the sensitivity of detecting rare TCR clonotypes and delineating a more comprehensive TCR repertoire. In contrast, TCRβ CDR3 DNA sequencing uses ImmunoSEQ platform (Adaptive Biotechnologies) which allows us to examine much more productive TCRβ CDR3 sequences and more in-depth characterization of the TCR repertoire. However, this platform does not allow the analysis of paired TCRα and TCRβ chains from a given T cell and we can only obtain TCRβ information. To better delineate the differences between these two techniques, we compared the total number of productive templates sequenced from either method, showing that more TCR sequences were obtained by ImmunoSEQ platform (**Supplemental Table 4**). Since we employed immunoSEQ platform to sequence only TCRβ chain, the caveat is that these top TCR clonotypes may indeed be mutually exclusive between R vs. NR CD8 TILs if we were able to detect the pairing TCRα chain for each corresponding TCRβ chain. Nonetheless, both single-cell TCR sequencing and TCRβ DNA sequencing showed that the top-expanded TCR clonotypes differed substantially between R vs. NR CD8 TILs, which also occurred in a recipient-specific manner, suggesting a highly individualized anti-tumor immune response.

We did not collect the DNA samples from control group in the current study; thus, we cannot perform ImmunoSEQ TCRβ sequencing of CD8 TILs from control group. However, we previously sequenced the TCR repertoire of CD8 TILs from control group (non-treated A223 tumor-bearing mice) (20) and R as well as NR group (anti-PD-L1 treated) (19) using single-cell TCR-sequencing. We found that the top-expanded TCR clonotypes differed substantially in a recipient-specific manner, indicating that each mouse expanded a different set of TCRs against the same A223 tumors (**Supplemental Figure 3**). These data are consistent with our conclusion that anti-tumor immune responses are highly individualized and different hosts may expand different TCR specificities against the same tumors, which may have important implications for developing personalized cancer immunotherapy.

Previous studies examined the TCR repertoires of HNSCCs in different settings (25–27); however, these studies have not identified clear biomarkers that would predict clinical responses to different therapies. We showed that cetuximab-treated HNSCC patients harbor dynamic changes of TCR repertoires correlative to therapeutic responses (28). This study also highlights the importance of data normalization for TCR repertoire analysis (28). A recent study showed that TCR clonality of TILs in metastatic melanoma is predictive for efficacy of PD-1 blockade immunotherapy (29). Consistently, another study of peripheral blood mononuclear cell (PBMC) samples from HNSCCs treated with anti-PD-1 and cetuximab suggests that certain characteristics of pre-treatment PBMC samples may predict the clinical response to combined treatment (30). However, so far, no studies have reported whether differences in TCR repertoires would predict or corelate to ICI responses in HNSCCs. Our study suggests that differences in the clonal frequency of top expanded TCR clonotypes may corelate to ICI responses. However, in our preclinical model, one SCC cell line was transplanted into WT B6 recipients, a scenario completely different from human HNSCCs which differ substantially in individual patients. How would our study be applicable to the human HNSCC setting? First, if HPV^+^ HNSCCs express HPV-related viral antigens, it is possible that different HNSCCs may contain HPV-reactive TCR clonotypes with varying clonal frequency, and the higher frequency of “useful” clones would certainly correlate with ICI responses, whereas the higher frequency of “futile” clones would correlate with ICI resistance. Second, in HPV^−^ HNSCCs, there are common tumor-associated antigens shared between different patients, and TCR clonotypes reactive to such common antigens may present with varying clonal frequency, which may correlate with different outcomes of ICI treatment. Overall, we propose that differential clonal frequencies of the TCR clonotypes that recognize the same antigen in different HNSCC patients may contribute to the heterogeneous outcomes of ICI treatment. Of course, this hypothesis needs to be tested with further studies requiring the utilization of antigen-specific model systems.

## Data availability statement

The data presented in the study are publicly available and deposited in GEO database (GEO accession number GSE227404). The data presented in the study are also deposited in the Adaptive ImmunoSEQ repository publicly available, https://clients.adaptivebiotech.com/pub/john-2023-fi. The immuneACCESS DOI is https://doi.org/10.21417/JJ2023FI.

## Ethics statement

The animal study was reviewed and approved by Institutional Animal Care and Use Committee of University of Colorado Anschutz Medical Campus (AMC) (Aurora, CO) and University of Pittsburgh (Pittsburgh, PA).

## Author contributions

Conceptualization, JW. Formal analysis, JJ, HG, ZH. Investigation, JJ, SC, MV. Methodology, RW. Supervision, ZC. Writing, JW, and JJ. All authors contributed to the article and approved the submitted version.
