# Supplementary material for: Host-specific differences in top-expanded TCR clonotypes correlate with divergent outcomes of anti-PD-L1 treatment in responders versus non-responders
Source: Front Immunol. 2023 Mar 27;14:1100520. doi: 10.3389/fimmu.2023.1100520 (PMC10084475; doi:10.3389/fimmu.2023.1100520)
Supplement: Supplementary file 1 [file Presentation_1.pdf]

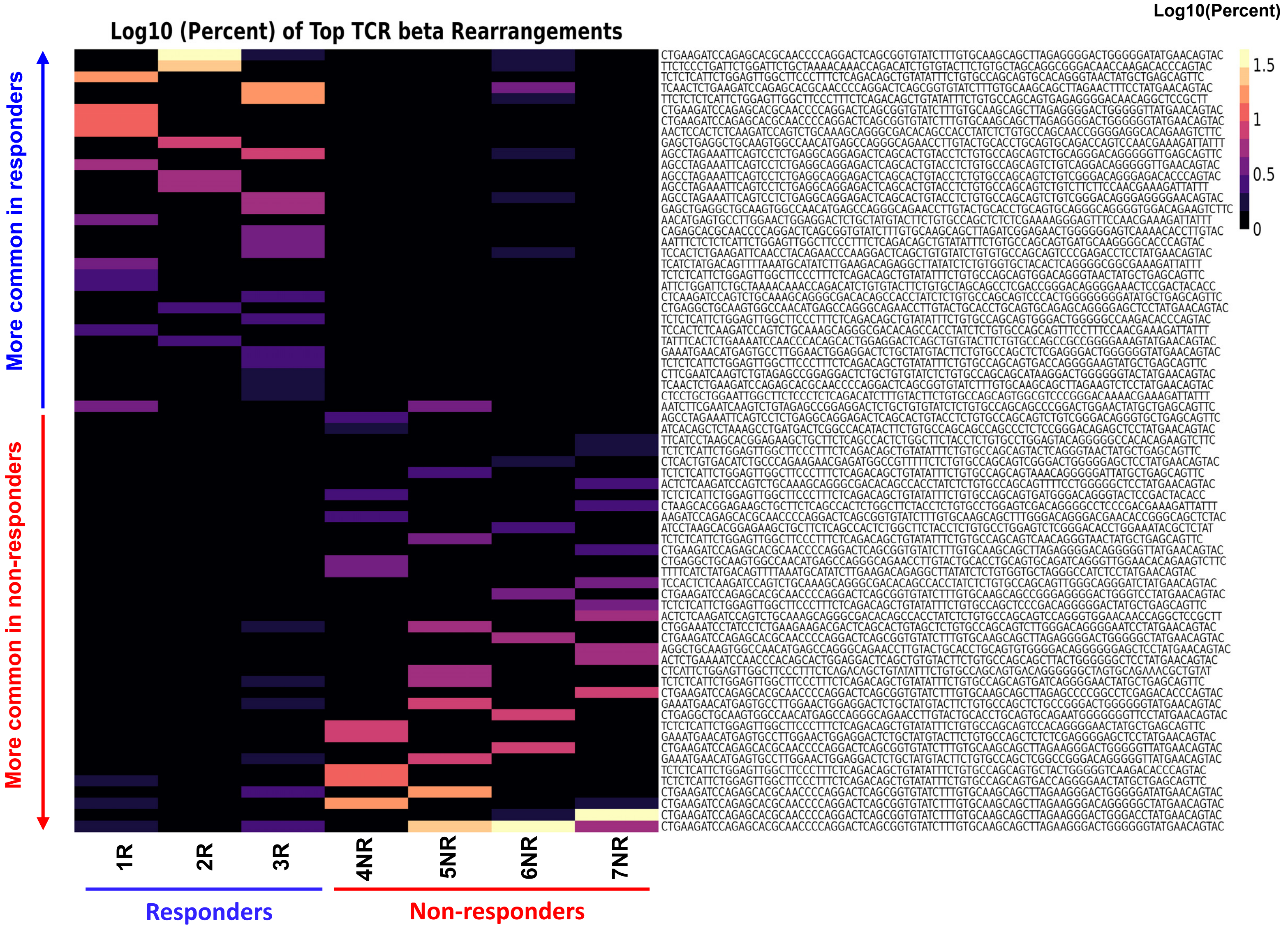

Supplemental Figure 1: Top TCRβ rearrangements appear to be mutually exclusive between responder (R) and non-responder (NR) CD8 T cells. Heatmap of TCRβ CDR3 DNA sequences. Top TCRβ CDR3 DNA sequences (abundance >1% of a given sample) were sorted by average abundance in R (1R, 2R, 3R) vs. average abundance in NR (4NR, 5NR, 6NR, 7NR). Cells with the same TCRβ CDR3 sequences were grouped together and colored according to the log10(percent) in each sample.

Supplemental Figure 1

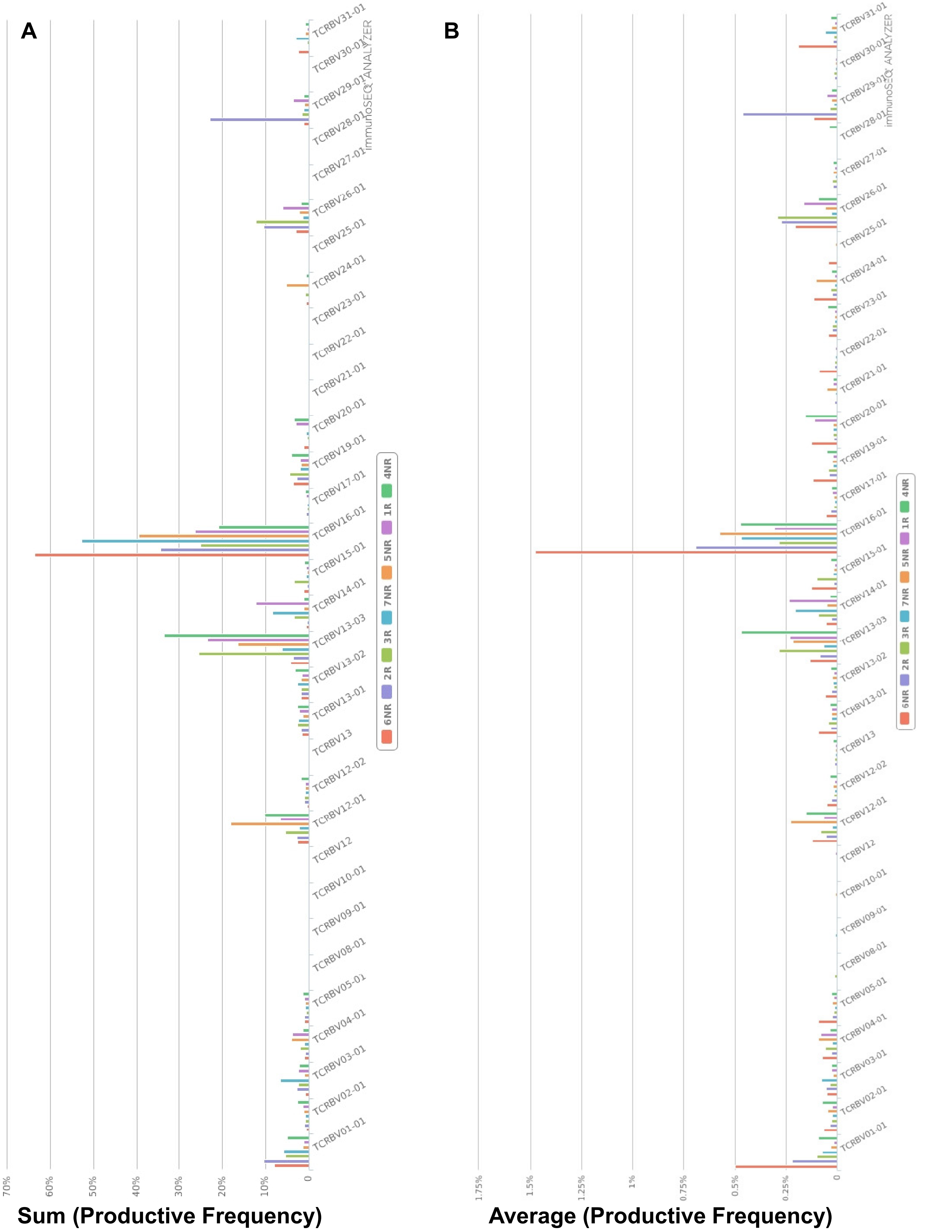

Supplemental Figure 2A, B

C

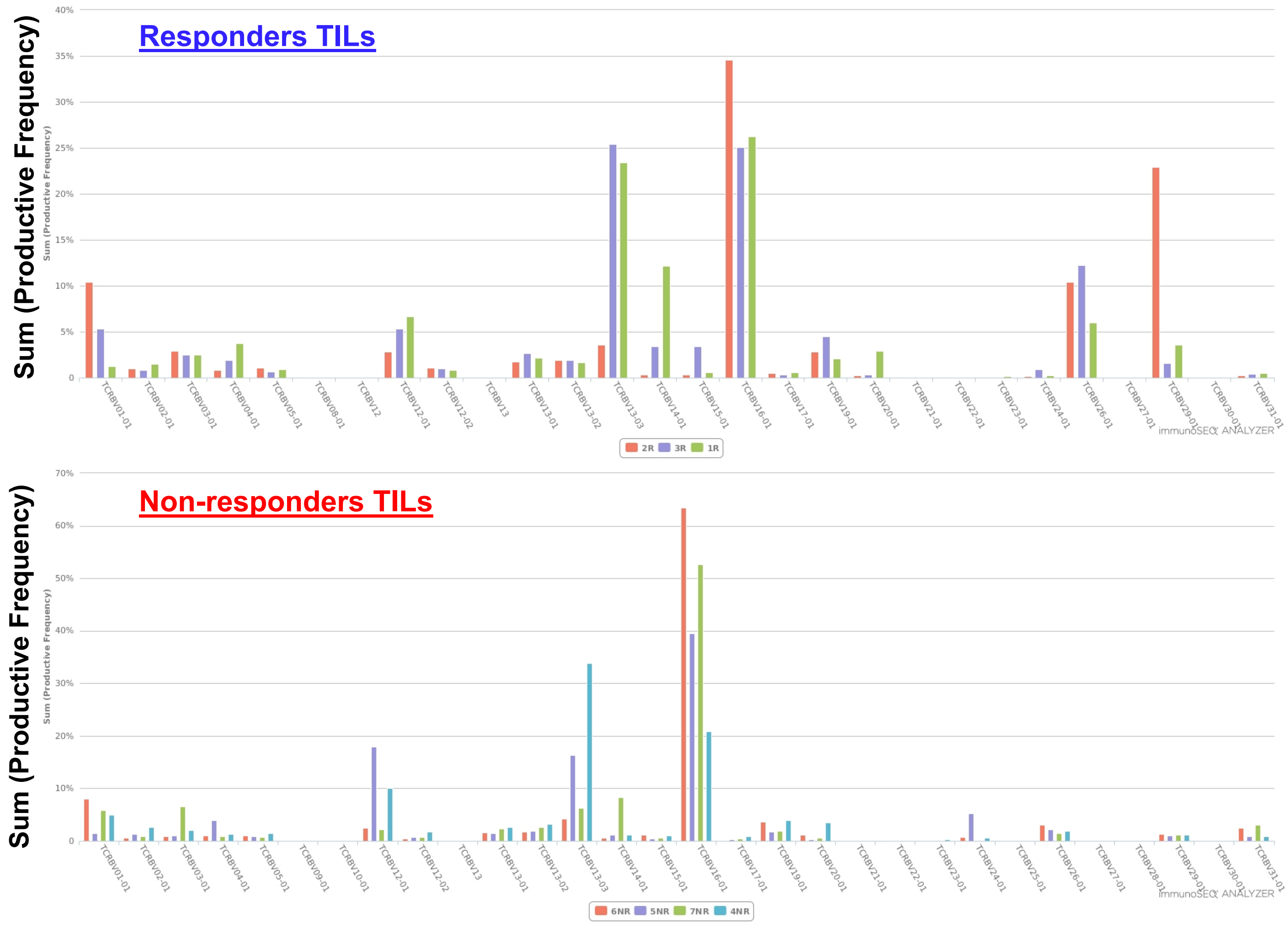

D

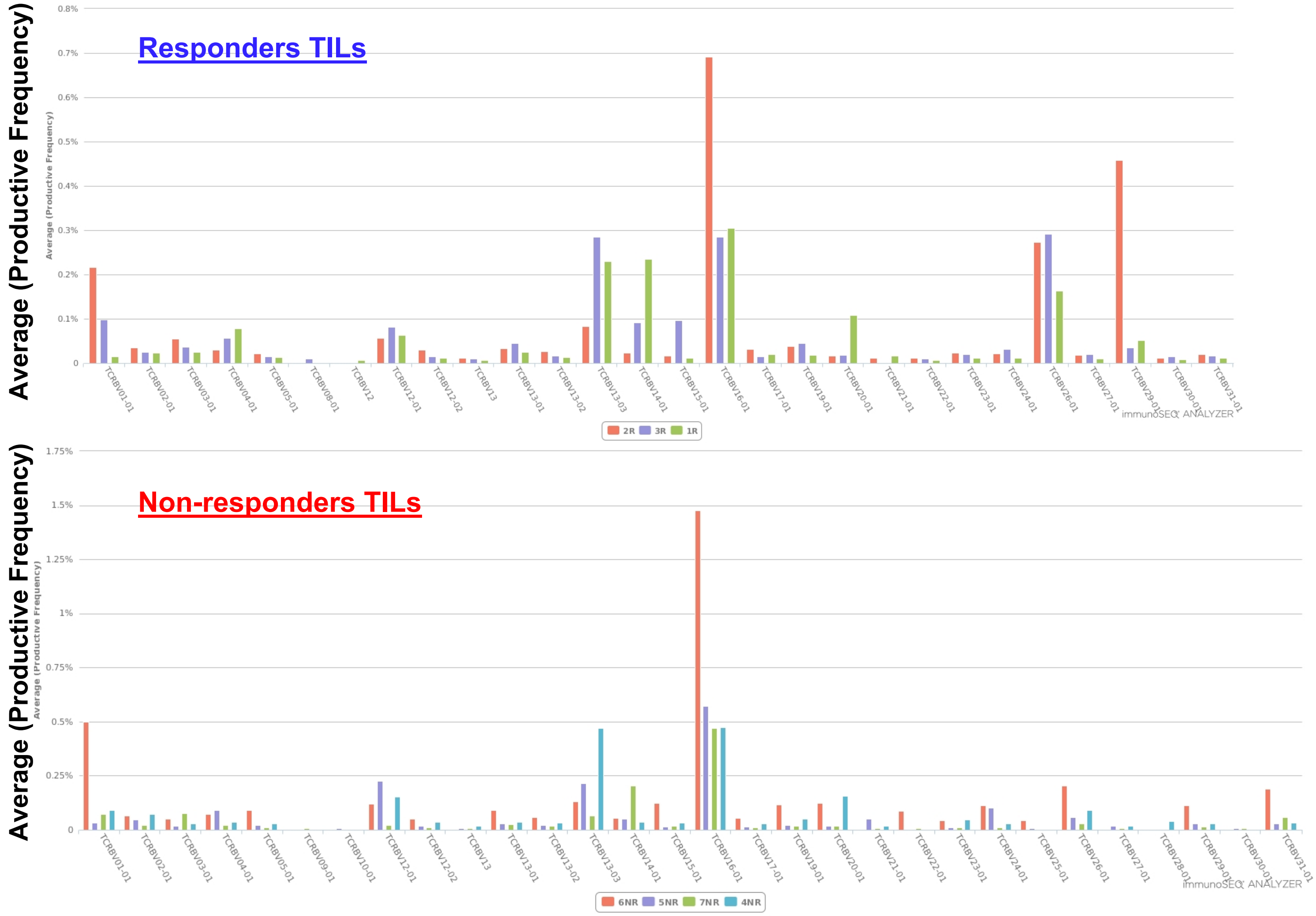

Supplemental Figure 2C, D

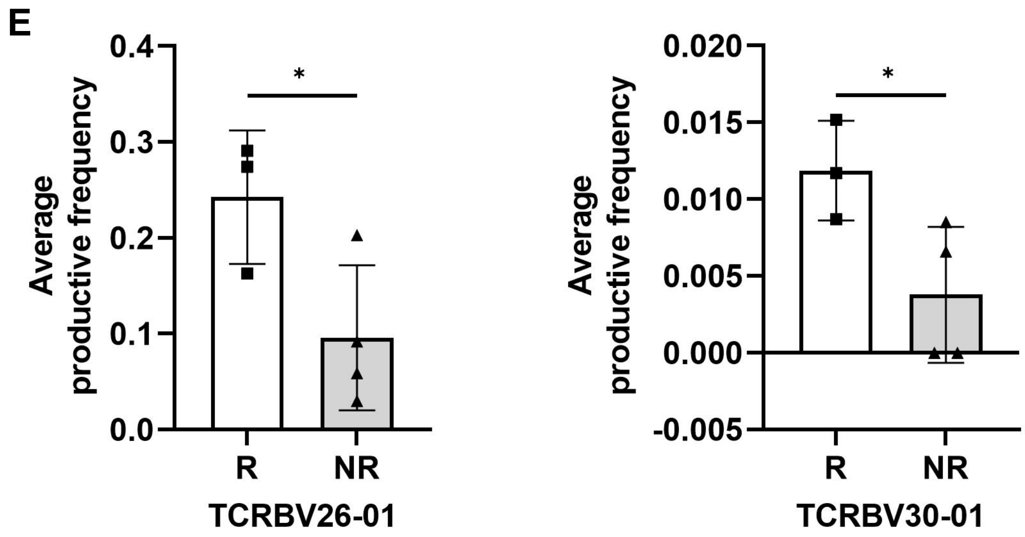

Supplemental Figure 2E

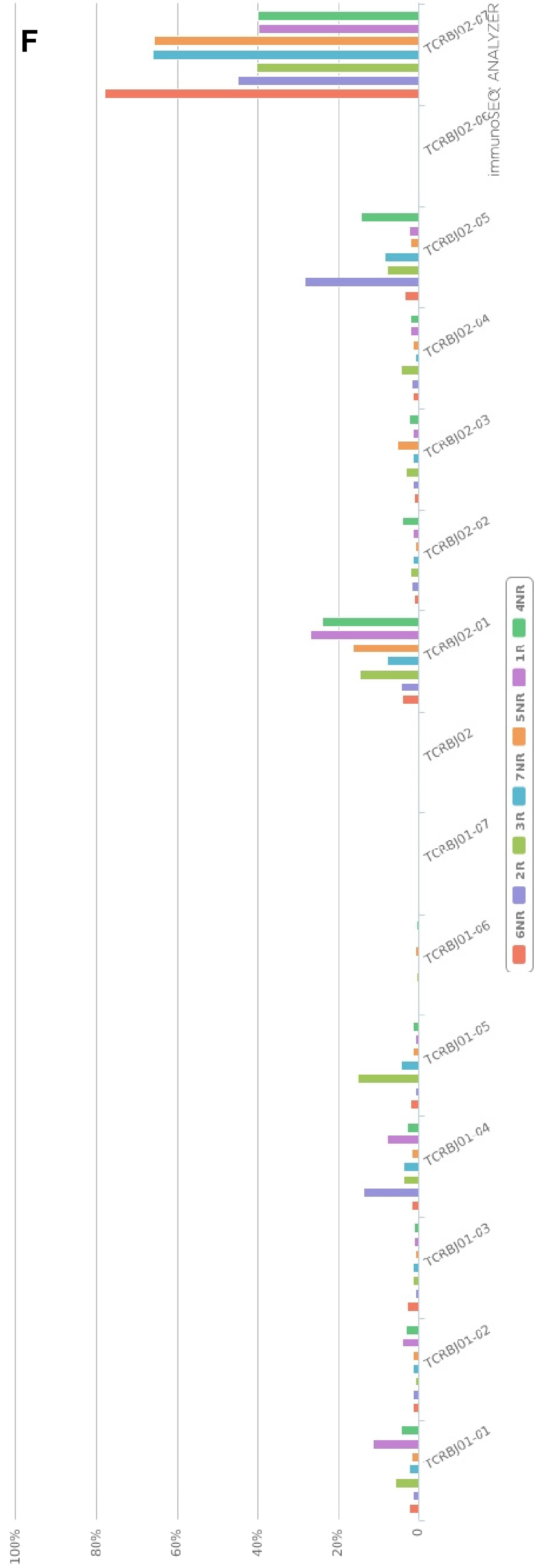

Sum (Productive Frequency)

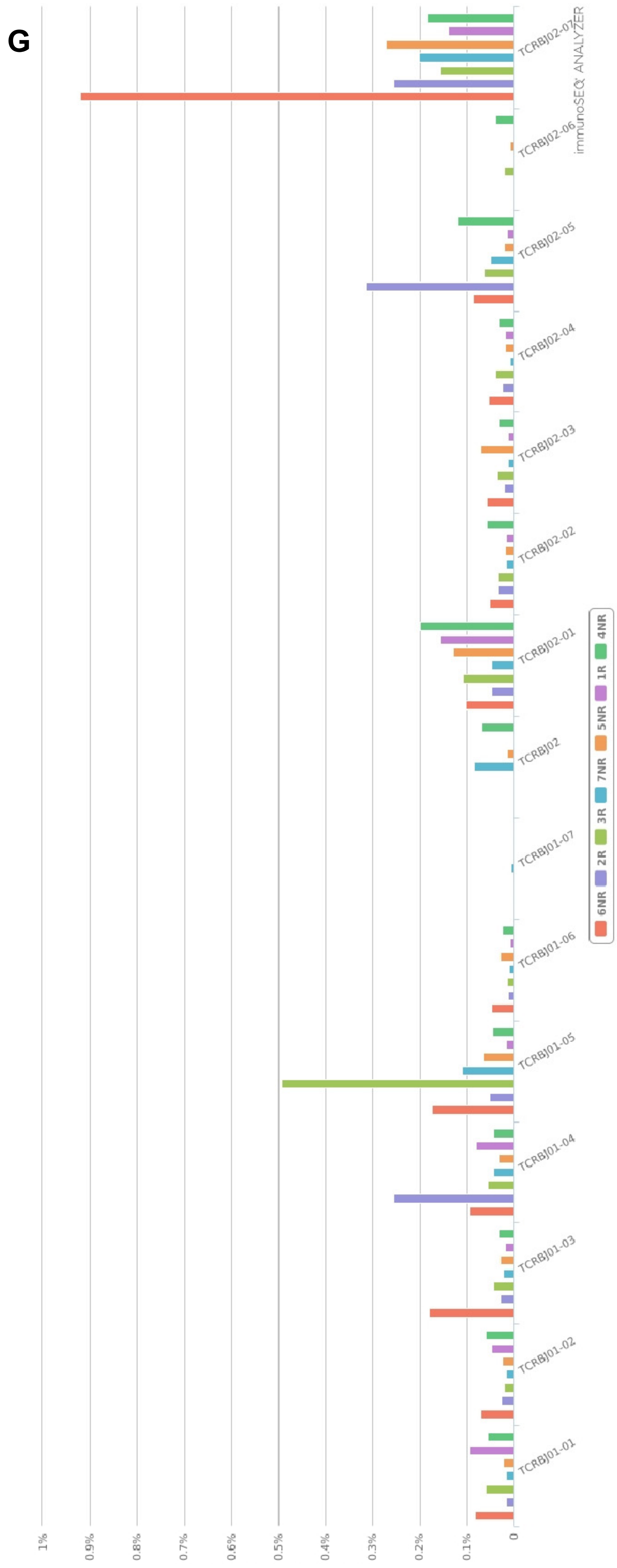

Average (Productive Frequency)

Supplemental Figure 2F, G

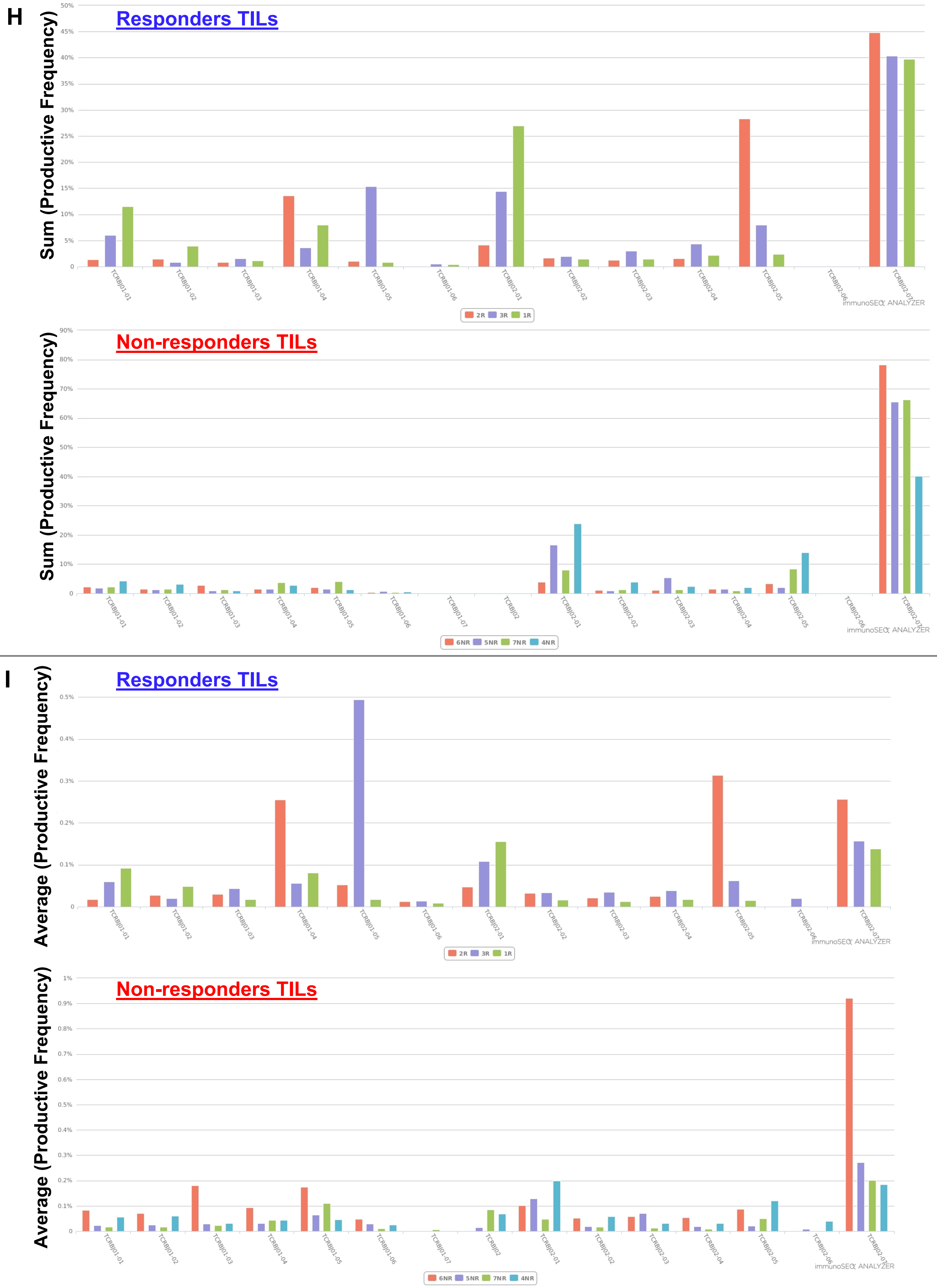

Supplemental Figure 2H, I

Supplemental Figure 2: Differential usage of V or J genes in responder (R) and non-responder (NR) CD8 T cells. (A-B) Histogram of the sum **(A)** or average **(B)** of productive frequency of a given TCRβ V gene usage in all the productive rearrangements of each TIL sample sequenced (1R-3R and 4NR-7NR) analyzed using the ImmunoSEQ analyzer. **(C-D)** Histogram of the sum **(C)** or average **(D)** of productive frequency of a given TCRβ V gene usage in all the productive rearrangements of each R or NR TIL sample (1R-3R vs. 4NR-7NR) analyzed using the ImmunoSEQ analyzer. Data are shown for R vs. NR separately (top vs. bottom in each panel) and for each R or NR sample individually within each plot. **(E)** Significant differences in the average productive frequency of V26-01 and V30-1 between R (n=3) and NR (n=4). **(F-G)** Histogram of the sum **(F)** or average **(G)** of productive frequency of a given TCRβ J gene usage in all the productive rearrangements of each TIL sample sequenced (1R-3R and 4NR-7NR) analyzed using the ImmunoSEQ analyzer. **(H-I)** Histogram of the sum **(H)** or average **(I)** of productive frequency of a given TCRβ J gene usage in all the productive rearrangements of each R or NR TIL sample (1R-3R vs. 4NR-7NR) analyzed using the ImmunoSEQ analyzer. Data are shown for R vs. NR separately (top vs. bottom in each panel) and for each R or NR sample individually within each plot.

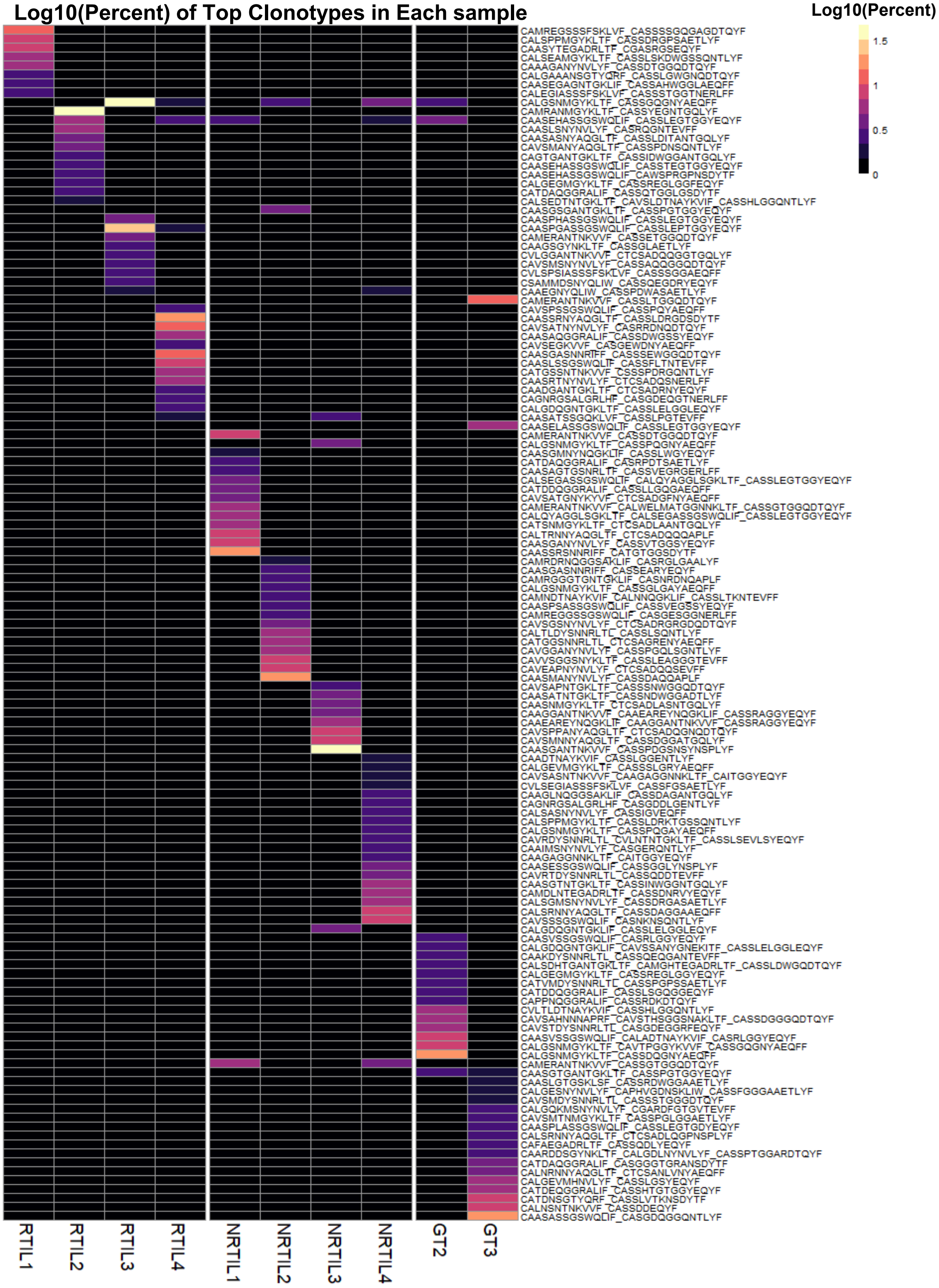

Supplemental Figure 3: Heatmap of top TCRαβ clonotypes (defined as >1% in any given sequenced TIL sample). Responder top TCR clonotypes were sorted by descending abundance (%) in RTIL1, RTIL2, RTIL3, and RTIL4, sequentially. The NR top TCR clonotypes were sorted by ascending abundance in NRTIL1, NRTIL2, NRTIL3, and NRTIL4, sequentially. The non-treated samples' top clonotypes were sorted by ascending abundance in GT2 and GT3 sequentially. Cells with a given clonotype are colored according to the log10(percent) in each sample. Single-cell TCR sequencing data of R (RTIL1, RTIL2, RTIL3, and RTIL4) samples and NR samples (NRTIL1, NRTIL2, NRTIL3, and NRTIL4) from our previous study (John et al., 2022) and single-cell TCR sequencing data for non-treated samples (GT1 and GT2) from previous study (Woolaver et al., 2021) were used for this analysis.

Supplemental Table 1: Seven samples sequenced by ImmunoSEQ assay

ID	Mouse	Tissue	Cells	Locus	Total templates	Productive templates	Total Rearrangements	Productive rearrangements
1R	Responder	Tumor	$CD8^+$	TCRB	18392	13807	2191	1409
2R	Responder	Tumor	$CD8^+$	TCRB	9460	8556	1201	766
3R	Responder	Tumor	$CD8^+$	TCRB	12726	9877	1771	1090
4NR	Non-responder	Tumor	$CD8^+$	TCRB	7019	5062	1501	948
5NR	Non-responder	Tumor	$CD8^+$	TCRB	14601	11741	1535	953
6NR	Non-responder	Tumor	$CD8^+$	TCRB	2861	2332	511	333
7NR	Non-responder	Tumor	$CD8^+$	TCRB	18642	15182	2220	1408

Supplemental Table 1: Seven samples sequenced by Immunoseq assay. Tumors were removed from 3 responder mice (1R, 2R, 3R) and 4 non-responder mice (4NR, 5NR, 6NR, 7NR); tumors were digested, and CD8 T cells were isolated followed by genomic DNA (gDNA) extraction. gDNA was subjected to ImmunoSeq TCR beta chain sequencing. Table displays the sample level information which includes the total number of templates captured, total number of productive templates, total number of rearrangements and productive rearrangements.

Supplemental Table 2: Detailed clonotype information for top 10 TCRβ rearrangements in each sample (Continued)

Sample	TCRβ CDR3 Amino_Acid	Rearrangement	TCRβV	TCRβD	TCRβJ	% of Sample
1R	CASSAQGNYAEQFF	TCTCTCATTCTGGAGTTGGCTTCCCTTTCTCAGACAGCTGTATATTTCTGTGCCAGCAGTGCACAGGGTAACTATGCTGAGCAGTTC	TCRBV13-03*01	TCRBD01-01*01	TCRBJ02-01*01	17.07
	CASSLEGTGGYEQYF	CTGAAGATCCAGAGCACGCAACCCCAGGACTCAGCGGTGTATCTTTGTGCAAGCAGCTTAGAGGGGACTGGGGGTTATGAACAGTAC	TCRBV16-01*01	TCRBD02-01*01	TCRBJ02-07*01	11.97
	CASSLEGTGGYEQYF	CTGAAGATCCAGAGCACGCAACCCCAGGACTCAGCGGTGTATCTTTGTGCAAGCAGCTTAGAGGGGACTGGGGGGTATGAACAGTAC	TCRBV16-01*01	TCRBD02-01*01	TCRBJ02-07*01	11.19
	CASNRGGTEVFF	AACTCCACTCTCAAGATCCAGTCTGCAAAGCAGGGCGACACAGCCACCTATCTCTGTGCCAGCAACCGGGGAGGCACAGAAGTCTTC	TCRBV14-01*01	unknown	TCRBJ01-01*01	9.46
	CASSLSGQGVEQYF	AGCCTAGAAATTCAGTCCTCTGAGGCAGGAGACTCAGCACTGTACCTCTGTGCCAGCAGTCTGTCAGGACAGGGGGTTGAACAGTAC	TCRBV26-01*01	TCRBD01-01*01	TCRBJ02-07*01	5.43
	CASSPDWNYAEQFF	AATCTTCGAATCAAGTCTGTAGAGCCGGAGGACTCTGCTGTGTATCTCTGTGCCAGCAGCCCGGACTGGAACTATGCTGAGCAGTTC	TCRBV04-01*01	TCRBD02-01*01	TCRBJ02-01*01	2.88
	CASSLEKGVSNERLFF	AACATGAGTGCCTTGGAACTGGAGGACTCTGCTATGTACTTCTGTGCCAGCTCTCTCGAAAAGGGAGTTTCCAACGAAAGATTATTT	TCRBV12-01*01	unknown	TCRBJ01-04*01	2.41
	CGATLRGGERLFF	TCATCTATGACAGTTTTAAATGCATATCTTGAAGACAGAGGCTTATATCTCTGTGGTGCTACACTCAGGGGCGGCGAAAGATTATTT	TCRBV20-01*01	TCRBD01-01*01	TCRBJ01-04*01	2.12
	CASSGQGNYAEQFF	TCTCTCATTCTGGAGTTGGCTTCCCTTTCTCAGACAGCTGTATATTTCTGTGCCAGCAGTGGACAGGGTAACTATGCTGAGCAGTTC	TCRBV13-03*01	TCRBD01-01*01	TCRBJ02-01*01	2.05
	CASSLDRDRGNSDYTF	ATTCTGGATTCTGCTAAAACAAACCAGACATCTGTGTACTTCTGTGCTAGCAGCCTCGACCGGGACAGGGGAAACTCCGACTACACC	TCRBV29-01*01	TCRBD01-01*01	TCRBJ01-02*01	1.91
2R	CASSLEGTGGYEQYF	CTGAAGATCCAGAGCACGCAACCCCAGGACTCAGCGGTGTATCTTTGTGCAAGCAGCTTAGAGGGGACTGGGGGATATGAACAGTAC	TCRBV16-01*01	TCRBD02-01*01	TCRBJ02-07*01	33.31
	CASRRDNQDTQYF	TTCTCCCTGATTCTGGATTCTGCTAAAACAAACCAGACATCTGTGTACTTCTGTGCTAGCAGGCGGGACAACCAAGACACCCAGTAC	TCRBV29-01*01	unknown	TCRBJ02-05*01	21.03
	CTCSADQSNERLFF	GAGCTGAGGCTGCAAGTGGCCAACATGAGCCAGGGCAGAACCTTGTACTGCACCTGCAGTGCAGACCAGTCCAACGAAAGATTATTT	TCRBV01-01*01	TCRBD01-01*01	TCRBJ01-04*01	7.49
	CASSLSGQGDTQYF	AGCCTAGAAATTCAGTCCTCTGAGGCAGGAGACTCAGCACTGTACCTCTGTGCCAGCAGTCTGTCGGGACAGGGAGACACCCAGTAC	TCRBV26-01*01	TCRBD01-01*01	TCRBJ02-05*01	4.63
	CASSLSSSNERLFF	AGCCTAGAAATTCAGTCCTCTGAGGCAGGAGACTCAGCACTGTACCTCTGTGCCAGCAGTCTGTCTTCTTCCAACGAAAGATTATTT	TCRBV26-01*01	unknown	TCRBJ01-04*01	4.25
	CTCSAEQGSSYEQYF	CTGAGGCTGCAAGTGGCCAACATGAGCCAGGGCAGAACCTTGTACTGCACCTGCAGTGCAGAGCAGGGGAGCTCCTATGAACAGTAC	TCRBV01-01*01	TCRBD01-01*01	TCRBJ02-07*01	1.69
	CASRRGKYEQYF	TATTTCACTCTGAAAATCCAACCCACAGCACTGGAGGACTCAGCTGTGTACTTCTGTGCCAGCCGCCGGGGAAAGTATGAACAGTAC	TCRBV03-01*01	unknown	TCRBJ02-07*01	1.48
	CASSHGTGGYEQYF	GAAATGAACATGAGTGCCTTGGAACTGGAGGACTCTGCTATGTACTTCTGTGCCAGCTCCCACGGGACAGGGGGCTATGAACAGTAC	TCRBV12-01*01	TCRBD01-01*01	TCRBJ02-07*01	0.97
	CASSENRNYEQYF	TTCTCTCTCATTCTGGAGTTGGCTTCCCTTTCTCAGACAGCTGTATATTTCTGTGCCAGCAGTGAGAACAGAAACTATGAACAGTAC	TCRBV13-03*01	TCRBD01-01*01	TCRBJ02-07*01	0.94
	CASSDAQNTLYF	AATTTCTCTCTCATTCTGGAGTTGGCTTCCCTTTCTCAGACAGCTGTATATTTCTGTGCCAGCAGTGATGCTCAAAACACCTTGTAC	TCRBV13-03*01	unknown	TCRBJ02-04*01	0.77
3R	CASSLELSYEQYF	TCAACTCTGAAGATCCAGAGCACGCAACCCCAGGACTCAGCGGTGTATCTTTGTGCAAGCAGCTTAGAACTTTCCTATGAACAGTAC	TCRBV16-01*01	TCRBD02-01*01	TCRBJ02-07*01	15.84
	CASSERGQQAPLF	TTCTCTCTCATTCTGGAGTTGGCTTCCCTTTCTCAGACAGCTGTATATTTCTGTGCCAGCAGTGAGAGGGGACAACAGGCTCCGCTT	TCRBV13-03*01	TCRBD01-01*01	TCRBJ01-05*01	14.66
	CASSLQGQGVEQFF	AGCCTAGAAATTCAGTCCTCTGAGGCAGGAGACTCAGCACTGTACCTCTGTGCCAGCAGTCTGCAGGGACAGGGGGTTGAGCAGTTC	TCRBV26-01*01	TCRBD01-01*01	TCRBJ02-01*01	6.44
	CASSLSGQGGEQYF	AGCCTAGAAATTCAGTCCTCTGAGGCAGGAGACTCAGCACTGTACCTCTGTGCCAGCAGTCTGTCGGGACAGGGAGGGGAACAGTAC	TCRBV26-01*01	TCRBD01-01*01	TCRBJ02-07*01	4.30
	CTCSAGQGWTEVFF	GAGCTGAGGCTGCAAGTGGCCAACATGAGCCAGGGCAGAACCTTGTACTGCACCTGCAGTGCAGGGCAGGGGTGGACAGAAGTCTTC	TCRBV01-01*01	TCRBD01-01*01	TCRBJ01-01*01	3.60
	CASSPETSYEQYF	TCCACTCTGAAGATTCAACCTACAGAACCCAAGGACTCAGCTGTGTATCTGTGTGCCAGCAGTCCCGAGACCTCCTATGAACAGTAC	TCRBV15-01*01	unknown	TCRBJ02-07*01	2.62
	CASSLDRRTGGSQNTLYF	CAGAGCACGCAACCCCAGGACTCAGCGGTGTATCTTTGTGCAAGCAGCTTAGATCGGAGAACTGGGGGGAGTCAAAACACCTTGTAC	TCRBV16-01*01	TCRBD02-01*01	TCRBJ02-04*01	2.59
	CASSDARGTQYF	AATTTCTCTCTCATTCTGGAGTTGGCTTCCCTTTCTCAGACAGCTGTATATTTCTGTGCCAGCAGTGATGCAAGGGGCACCCAGTAC	TCRBV13-03*01	TCRBD01-01*01	TCRBJ02-05*01	2.52
	CASSPTGGGYAEQFF	CTCAAGATCCAGTCTGCAAAGCAGGGCGACACAGCCACCTATCTCTGTGCCAGCAGTCCCACTGGGGGGGGATATGCTGAGCAGTTC	TCRBV14-01*01	TCRBD02-01*01	TCRBJ02-01*01	1.84
	CASSGTGGQDTQYF	TCTCTCATTCTGGAGTTGGCTTCCCTTTCTCAGACAGCTGTATATTTCTGTGCCAGCAGTGGGACTGGGGGCCAAGACACCCAGTAC	TCRBV13-03*01	TCRBD02-01*01	TCRBJ02-05*01	1.79

Supplemental Table 2: Detailed clonotype information for top 10 TCRβ rearrangements in each sample

Sample	TCRβ CDR3 Amino_Acid	Rearrangement	TCRβV	TCRβD	TCRβJ	% of Sample
4NR	CASSLEGTGGYEQYF	CTGAAGATCCAGAGCACGCAACCCCAGGACTCAGCGGTGTATCTTTGTGCAAGCAGCTTAGAAGGGACAGGGGGCTATGAACAGTAC	TCRBV16-01*01	TCRBD01-01*01	TCRBJ02-07*01	17.25
	CASSDQGNYAEQFF	TCTCTCATTCTGGAGTTGGCTTCCCTTTCTCAGACAGCTGTATATTTCTGTGCCAGCAGTGACCAGGGGAACTATGCTGAGCAGTTC	TCRBV13-03*01	TCRBD01-01*01	TCRBJ02-01*01	11.08
	CASSATGGQDTQYF	TCTCTCATTCTGGAGTTGGCTTCCCTTTCTCAGACAGCTGTATATTTCTGTGCCAGCAGTGCTACTGGGGGTCAAGACACCCAGTAC	TCRBV13-03*01	TCRBD02-01*01	TCRBJ02-05*01	10.49
	CASSPQGNYAEQFF	TCTCTCATTCTGGAGTTGGCTTCCCTTTCTCAGACAGCTGTATATTTCTGTGCCAGCAGTCCACAGGGGAACTATGCTGAGCAGTTC	TCRBV13-03*01	TCRBD01-01*01	TCRBJ02-01*01	7.31
	CASSLEGSSYEQYF	GAAATGAACATGAGTGCCTTGGAACTGGAGGACTCTGCTATGTACTTCTGTGCCAGCTCTCTCGAGGGGAGCTCCTATGAACAGTAC	TCRBV12-01*01	TCRBD01-01*01	TCRBJ02-07*01	7.25
	CGARAISYEQYF	TTTTCATCTATGACAGTTTTAAATGCATATCTTGAAGACAGAGGCTTATATCTCTGTGGTGCTAGGGCCATCTCCTATGAACAGTAC	TCRBV20-01*01	TCRBD01-01*01	TCRBJ02-07*01	2.67
	CTCSADQGWNTEVFF	CTGAGGCTGCAAGTGGCCAACATGAGCCAGGGCAGAACCTTGTACTGCACCTGCAGTGCAGATCAGGGTTGGAACACAGAAGTCTTC	TCRBV01-01*01	TCRBD01-01*01	TCRBJ01-01*01	2.43
	CASSFGTGTNTGQLYF	AAGATCCAGAGCACGCAACCCCAGGACTCAGCGGTGTATCTTTGTGCAAGCAGCTTTGGGACAGGGACGAACACCGGGCAGCTCTAC	TCRBV16-01*01	TCRBD01-01*01	TCRBJ02-02*01	1.64
	CASSDGTGYSDYTF	TCTCTCATTCTGGAGTTGGCTTCCCTTTCTCAGACAGCTGTATATTTCTGTGCCAGCAGTGATGGGACAGGGTACTCCGACTACACC	TCRBV13-03*01	TCRBD01-01*01	TCRBJ01-02*01	1.40
	CASSLSGQGAEQFF	AGCCTAGAAATTCAGTCCTCTGAGGCAGGAGACTCAGCACTGTACCTCTGTGCCAGCAGTCTGTCGGGACAGGGTGCTGAGCAGTTC	TCRBV26-01*01	TCRBD01-01*01	TCRBJ02-01*01	1.19
5NR	CASSLEGTGGYEQYF	CTGAAGATCCAGAGCACGCAACCCCAGGACTCAGCGGTGTATCTTTGTGCAAGCAGCTTAGAAGGGACTGGGGGGTATGAACAGTAC	TCRBV16-01*01	TCRBD02-01*01	TCRBJ02-07*01	23.11
	CASSLEGTGGYEQYF	CTGAAGATCCAGAGCACGCAACCCCAGGACTCAGCGGTGTATCTTTGTGCAAGCAGCTTAGAAGGGACTGGGGGATATGAACAGTAC	TCRBV16-01*01	TCRBD02-01*01	TCRBJ02-07*01	14.84
	CASSAGTGGYEQYF	GAAATGAACATGAGTGCCTTGGAACTGGAGGACTCTGCTATGTACTTCTGTGCCAGCTCGGCCGGGACAGGGGGTTATGAACAGTAC	TCRBV12-01*01	TCRBD01-01*01	TCRBJ02-07*01	8.25
	CASSAGTGGYEQYF	GAAATGAACATGAGTGCCTTGGAACTGGAGGACTCTGCTATGTACTTCTGTGCCAGCTCTGCCGGGACTGGGGGGTATGAACAGTAC	TCRBV12-01*01	TCRBD02-01*01	TCRBJ02-07*01	6.98
	CASSDQGNYAEQFF	TCTCTCATTCTGGAGTTGGCTTCCCTTTCTCAGACAGCTGTATATTTCTGTGCCAGCAGTGATCAGGGGAACTATGCTGAGCAGTTC	TCRBV13-03*01	TCRBD01-01*01	TCRBJ02-01*01	5.26
	CASSDRGASAETLYF	CTCATTCTGGAGTTGGCTTCCCTTTCTCAGACAGCTGTATATTTCTGTGCCAGCAGTGACAGGGGGGCTAGTGCAGAAACGCTGTAT	TCRBV13-03*01	unknown	TCRBJ02-03*01	4.48
	CASSLGTGESYEQYF	CTGGAAATCCTATCCTCTGAAGAAGACGACTCAGCACTGTAGCTCTGTGCCAGCAGTCTTGGGACAGGGGAATCCTATGAACAGTAC	TCRBV24-01*02	TCRBD01-01*01	TCRBJ02-07*01	4.00
	CASSPDWNYAEQFF	AATCTTCGAATCAAGTCTGTAGAGCCGGAGGACTCTGCTGTGTATCTCTGTGCCAGCAGCCCGGACTGGAACTATGCTGAGCAGTTC	TCRBV04-01*01	TCRBD02-01*01	TCRBJ02-01*01	3.31
	CASSQQGNYAEQFF	TCTCTCATTCTGGAGTTGGCTTCCCTTTCTCAGACAGCTGTATATTTCTGTGCCAGCAGTCAACAGGGTAACTATGCTGAGCAGTTC	TCRBV13-03*01	TCRBD01-01*01	TCRBJ02-01*01	2.21
	CASSKQGDYAEQFF	TCTCTCATTCTGGAGTTGGCTTCCCTTTCTCAGACAGCTGTATATTTCTGTGCCAGCAGTAAACAGGGGGATTATGCTGAGCAGTTC	TCRBV13-03*01	TCRBD01-01*01	TCRBJ02-01*01	1.35
6NR	CASSLEGTGGYEQYF	CTGAAGATCCAGAGCACGCAACCCCAGGACTCAGCGGTGTATCTTTGTGCAAGCAGCTTAGAAGGGACTGGGGGGTATGAACAGTAC	TCRBV16-01*01	TCRBD02-01*01	TCRBJ02-07*01	42.54
	CASSLEGTGGYEQYF	CTGAAGATCCAGAGCACGCAACCCCAGGACTCAGCGGTGTATCTTTGTGCAAGCAGCTTAGAAGGGACTGGGGGTTATGAACAGTAC	TCRBV16-01*01	TCRBD02-01*01	TCRBJ02-07*01	7.25
	CTCSAEWGGSYEQYF	CTGAGGCTGCAAGTGGCCAACATGAGCCAGGGCAGAACCTTGTACTGCACCTGCAGTGCAGAATGGGGGGGTTCCTATGAACAGTAC	TCRBV01-01*01	TCRBD02-01*01	TCRBJ02-07*01	6.82
	CASSLEGTGGYEQYF	CTGAAGATCCAGAGCACGCAACCCCAGGACTCAGCGGTGTATCTTTGTGCAAGCAGCTTAGAGGGGACTGGGGGCTATGAACAGTAC	TCRBV16-01*01	TCRBD02-01*01	TCRBJ02-07*01	3.69
	CASSREGTGSYEQYF	CTGAAGATCCAGAGCACGCAACCCCAGGACTCAGCGGTGTATCTTTGTGCAAGCAGCCGGGAGGGGACTGGGTCCTATGAACAGTAC	TCRBV16-01*01	TCRBD02-01*01	TCRBJ02-07*01	2.83
	CASSLELSYEQYF	TCAACTCTGAAGATCCAGAGCACGCAACCCCAGGACTCAGCGGTGTATCTTTGTGCAAGCAGCTTAGAACTTTCCTATGAACAGTAC	TCRBV16-01*01	TCRBD02-01*01	TCRBJ02-07*01	2.23
	CAWSLGTPGNTLYF	ATCCTAAGCACGGAGAAGCTGCTTCTCAGCCACTCTGGCTTCTACCTCTGTGCCTGGAGTCTCGGGACACCTGGAAATACGCTCTAT	TCRBV31-01*01	TCRBD01-01*01	TCRBJ01-03*01	1.67
	CASSERGQQAPLF	TTCTCTCTCATTCTGGAGTTGGCTTCCCTTTCTCAGACAGCTGTATATTTCTGTGCCAGCAGTGAGAGGGGACAACAGGCTCCGCTT	TCRBV13-03*01	TCRBD01-01*01	TCRBJ01-05*01	1.07
	CASSRDWGSSYEQYF	CTCACTGTGACATCTGCCCAGAAGAACGAGATGGCCGTTTTTCTCTGTGCCAGCAGTCGGGACTGGGGGAGCTCCTATGAACAGTAC	TCRBV19-01*01	TCRBD02-01*01	TCRBJ02-07*01	1.07
	CASSLQGQGVEQFF	AGCCTAGAAATTCAGTCCTCTGAGGCAGGAGACTCAGCACTGTACCTCTGTGCCAGCAGTCTGCAGGGACAGGGGGTTGAGCAGTTC	TCRBV26-01*01	TCRBD01-01*01	TCRBJ02-01*01	0.69
7NR	CASSLEGTGTYEQYF	CTGAAGATCCAGAGCACGCAACCCCAGGACTCAGCGGTGTATCTTTGTGCAAGCAGCTTAGAAGGGACTGGGACCTATGAACAGTAC	TCRBV16-01*01	TCRBD02-01*01	TCRBJ02-07*01	37.56
	CASSLEPRPRDTQYF	CTGAAGATCCAGAGCACGCAACCCCAGGACTCAGCGGTGTATCTTTGTGCAAGCAGCTTAGAGCCCCGGCCTCGAGACACCCAGTAC	TCRBV16-01*01	unknown	TCRBJ02-05*01	6.07
	CASSLLGGSYEQYF	ACTCTGAAAATCCAACCCACAGCACTGGAGGACTCAGCTGTGTACTTCTGTGCCAGCAGCTTACTGGGGGGCTCCTATGAACAGTAC	TCRBV03-01*01	TCRBD02-01*01	TCRBJ02-07*01	4.23
	CTCSVGTGGSSYEQYF	AGGCTGCAAGTGGCCAACATGAGCCAGGGCAGAACCTTGTACTGCACCTGCAGTGTGGGGACAGGGGGGAGCTCCTATGAACAGTAC	TCRBV01-01*01	TCRBD01-01*01	TCRBJ02-07*01	4.05
	CASSLEGTGGYEQYF	CTGAAGATCCAGAGCACGCAACCCCAGGACTCAGCGGTGTATCTTTGTGCAAGCAGCTTAGAAGGGACTGGGGGGTATGAACAGTAC	TCRBV16-01*01	TCRBD02-01*01	TCRBJ02-07*01	3.65
	CASSPGWNNQAPLF	ACTCTCAAGATCCAGTCTGCAAAGCAGGGCGACACAGCCACCTATCTCTGTGCCAGCAGTCCAGGGTGGAACAACCAGGCTCCGCTT	TCRBV14-01*01	TCRBD01-01*01	TCRBJ01-05*01	3.57
	CASSRQGDYAEQFF	TCTCTCATTCTGGAGTTGGCTTCCCTTTCTCAGACAGCTGTATATTTCTGTGCCAGCTCCCGACAGGGGGACTATGCTGAGCAGTTC	TCRBV13-03*01	TCRBD01-01*01	TCRBJ02-01*01	3.42
	CASSWAGIYEQYF	TCCACTCTCAAGATCCAGTCTGCAAAGCAGGGCGACACAGCCACCTATCTCTGTGCCAGCAGTTGGGCAGGGATCTATGAACAGTAC	TCRBV14-01*01	TCRBD01-01*01	TCRBJ02-07*01	2.94
	CASSLEGTGGYEQYF	CTGAAGATCCAGAGCACGCAACCCCAGGACTCAGCGGTGTATCTTTGTGCAAGCAGCTTAGAGGGGACAGGGGGTTATGAACAGTAC	TCRBV16-01*01	TCRBD01-01*01	TCRBJ02-07*01	1.94
	CAWSRQGPPDERLFF	CTAAGCACGGAGAAGCTGCTTCTCAGCCACTCTGGCTTCTACCTCTGTGCCTGGAGTCGACAGGGGCCTCCCGACGAAAGATTATTT	TCRBV31-01*01	TCRBD01-01*01	TCRBJ01-04*01	1.45

Supplemental Table 2. Detailed clonotype information for top 10 TCRβ rearrangements in each sample. CD8 T cells from each sample were grouped into clones by identical nucleotide sequence of the TCRβ CDR3 chain. The top 10 rearrangements by abundance in each sample are shown, with their corresponding CDR3 amino acid sequence, nucleotide sequence (rearrangement), V, D, and J gene and allele usage, and percent (% = the number of cells with each rearrangement / the number of total cells sequenced for a given sample).

Supplemental Table 3: TCRβ CDR3 sequences shared in 7 TIL samples

ID Number	TCRβ CDR3	1R	2R	3R	4NR	5NR	6NR	7NR
**1**	CASSLEGTGGYEQYF	24.676	33.462	3.402	17.404	38.123	54.674	7.509
**2**	CASSGTGGQDTQYF	0.036	0.105	1.822	0.040	0.009	0.172	0.283
**3**	CASSPGTGGYEQYF	0.167	0.140	0.071	0.099	1.244	0.472	0.020
**4**	CASSLSGQGAEQFF	0.167	0.117	0.030	1.185	0.136	0.214	0.013
**5**	CASSLGTGGYEQYF	0.014	0.409	0.172	0.040	0.034	0.129	0.033

Supplemental Table 3: TCRβ CDR3 amino acid sequences shared in 7 TIL samples. TCRβ CDR3 amino acid sequences shared between all seven TIL samples were evaluated. Five shared TCRβ CDR3 sequences with percent (% = the number of cells with each TCRβ CDR3 amino acid sequence / the number of total cells sequenced for a given sample) in each sample are shown.

Supplemental Table 4: Samples sequenced by Single-cell TCR sequencing (10× Genomics) or immunoSEQ TCRβ DNA sequencing (Adaptive Biotechnologies)

Single-cell TCR sequencing		
**ID**	**Mouse**	**#TCR**
RTIL1	Responder #1	2810
RTIL2	Responder #2	5175
RTIL3	Responder #3	1777
RTIL4	Responder #4	3056
NRTIL1	Non-responder #1	615
NRTIL2	Non-responder #2	1248
NRTIL3	Non-responder #3	1885
NRTIL4	Non-responder #4	3043

ImmunoSEQ TCRβ DNA sequencing		
**ID**	**Mouse**	**#TCR**
1R	Responder #1	13807
2R	Responder #2	8556
3R	Responder #3	9877
4NR	Non-responder #1	5062
5NR	Non-responder #2	11741
6NR	Non-responder #3	2332
7NR	Non-responder #4	15182

Supplemental Table 4: Comparison of the number of productive TCRs sequenced by Single-cell TCR sequencing (10× Genomics) or immunoSEQ TCRβ DNA sequencing (Adaptive Biotechnologies). CD8 T cells isolated from the tumors of 4 responding and 4 non-responding mice (John J, 2022) were subjected to 10× Genomics TCR VDJ sequencing. CD8 T cells isolated from the tumors of 3 responding mice and 4 non-responding mice (in the current study) were subjected to immunoSEQ TCRβ DNA sequencing.
